# Rehabilitation Considerations for Very Young Children with Severe Oligodontia due to Ectodermal Dysplasia: Report of Three Clinical Cases with a 2-Year Follow-Up

**DOI:** 10.1155/2022/9925475

**Published:** 2022-03-22

**Authors:** Kyriaki Seremidi, Antigoni Markouli, Andreas Agouropoulos, Nick Polychronakis, Sotiria Gizani

**Affiliations:** ^1^Department of Pediatric Dentistry, School of Dentistry, National and Kapodistrian University of Athens, Greece; ^2^Department of Prosthodontics, School of Dentistry, National and Kapodistrian University of Athens, Greece

## Abstract

**Introduction:**

Management of oligodontia can be complicated and requires multidiscipline care, involving a wide spectrum of interventions. The aim of this report is to describe the challenges of oral rehabilitation of three very young children with oligodontia. *Report*. Three preschool aged Caucasian males, diagnosed with ectodermal dysplasia, were treated with interim removable dentures in order to replace missing teeth, reclaim vertical dimension, and improve function and aesthetics. The main challenges faced were patient cooperation, dental and skeletal characteristics, and parental expectations. Two years post-treatment, both patients and parents reported excellent adaptation to prosthesis and satisfaction with aesthetics.

**Conclusion:**

Rehabilitation of oligodontia may be challenging due to accompanying oral findings, dentofacial growth considerations, and behavioral issues. Establishment of good rapport between patients, parents, and clinician is the key for the success of the treatment even with the use of nonpharmacological behavioral management techniques.

## 1. Introduction

Oligodontia is defined as the absence of six or more teeth and is one of the most common dental developmental anomalies, with an estimated prevalence of 0.25% in the general population [1]. It can be sporadic, congenital, or syndromic, with ectodermal dysplasia (ED) being most commonly associated with it [2]. ED is a group of rare genetic disorders characterized by abnormalities in at least two ectodermal structures such as skin, hair, nails, teeth, and glands and has three distinct features: hypohidrosis, hypotrichosis, and oligodontia or anodontia [2–5].

Manifestations in the head and neck region include frontal bossing, saddle nose, reduced lower facial height, and class III skeletal relationship [5, 6]. Oral features include oligodontia, delayed eruption, and microdontia, with mild cases showing defects on maxillary lateral incisors and more severe cases showing defects of the entire dentition [2, 3, 7, 8]. Other features reported include retention of primary teeth, infraocclusion, taurodontism, and failure of alveolar bone growth [3, 9].

Management of oligodontia requires complex multidiscipline care and involves a wide spectrum of interventions, ranging from simple space maintenance to removable or fixed prostheses and implants [10]. At present, there is no standardized approach for oligodontia rehabilitation in paediatric patients [11]. Oral rehabilitation can be complicated due to absence of primary and permanent teeth and anatomical abnormalities of the existing teeth [3]. This, in association to reduced growth rate of the alveolar ridges, that results in a “knife-edge” ridge with decreased height, renders treatment planning more demanding [2, 6].

Oligodontia has a direct impact on patients' oral health-related quality of life (OHRQoL). These patients face physical, emotional, social, and behavioral challenges that can affect their self-esteem, and they, therefore, need regular follow-ups and continuous reassessment for lifetime, increasing stress and financial burden [12]. Evidence on functional implications, is inconclusive, with some studies showing a direct impact, mainly on mastication and speech [13–15] and others no significant effect [16].

The aim of this report is to describe the challenges faced in the oral rehabilitation of three preschool aged children with severe oligodontia due to ED.

## 2. Case One

A 3.5-year-old Caucasian boy, previously diagnosed with ED, was referred to the Postgraduate Clinic of the Paediatric Dentistry Department (NKUA) by a general dentist due to hismother's strong desire for restoring aesthetics by replacing his missing teeth.

**Figure 1:**
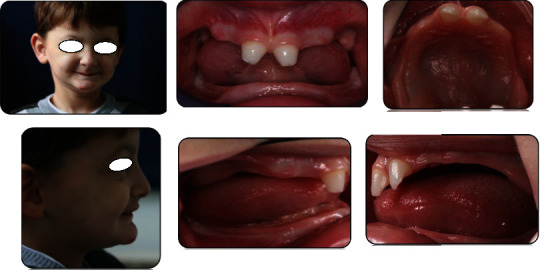
Initial extraoral and intraoral examination presenting main features of ectodermal dysplasia but normal hair and skin.

Extraoral examination revealed decreased lower facial height, rings under the eyes, everted nose, and protrusion of the chin. Unlike ectodermal dysplasia's common characteristics, patients presented normal skin and hair texture (Figure 1). Intraoral examination showed severe oligodontia with the only teeth present being the two upper central primary incisors that presented an irregular conical shape. Alveolar ridges of both arches were underdeveloped and flat with reduced height (Figure 1).

**Figure 2:**
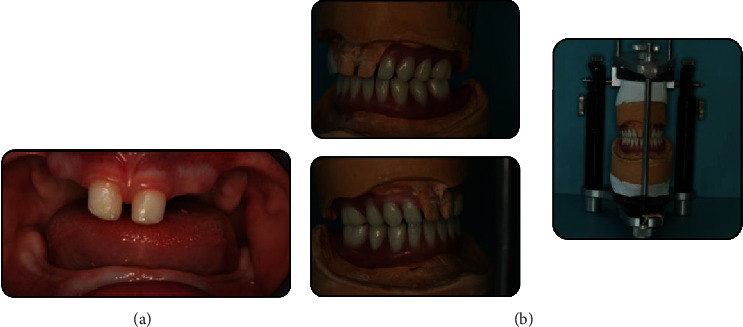
Different treatment stages: (a) composite build-ups and (b) dentures wax-up.

Treatment planning, as in all similar cases, was discussed and decided with the collaboration of the Departments of Paediatric Dentistry, Orthodontics, and Prosthodontics. Initially, an individualized preventive program was applied with emphasis on the oral hygiene, which was neglected by the parents, as it was not considered important. Upper primary incisors were restored with composite build-ups (Figure 2(a)) in order to obtain a better anatomical form to allow for adequate prosthetic retention. Rehabilitation was completed with the construction of an interim removable partial denture for the upper arch and a complete denture for the lower arch. Acrylic resin bases with wax record blocks (rims) were constructed to allow jaw relation recording (Figure 2(b)). The establishment of the occlusal vertical dimension was made applying the Willis method, and the anteroposterior position of the maxilla in relation to the mandible was registered with the latter in centric position.

**Figure 3:**
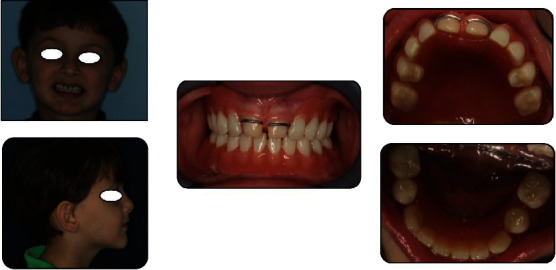
Extraoral and intraoral photographs after treatment completion.

The upper interim removable partial denture was constructed with wrought-wire clasps on the incisors for better retention. Both prostheses extended up to the region of second primary molars, to allow space for permanent molars to erupt. Vertical dimension and lower facial height were restored (Figure 3).

## 3. Case Two

A 3.5-year-old Caucasian boy presented in our Department with a chief complaint of multiple missing teeth. He had been diagnosed with x-linked ED, since birth, with no other medical problems, while his mother and his older brother had been also diagnosed with the same disorder.

**Figure 4:**
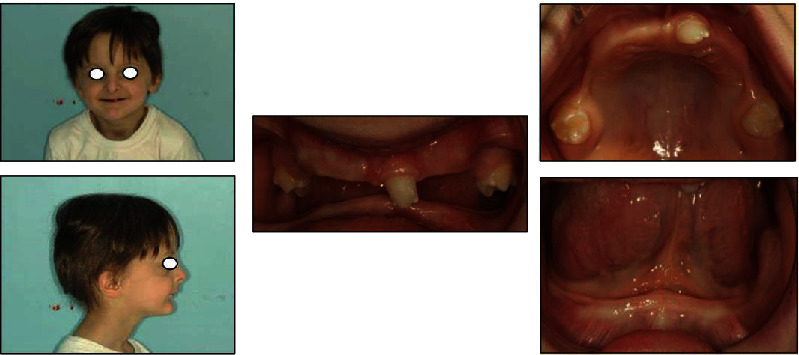
Initial extraoral and intraoral examination showing main clinical features of ED.

Extraoral examination revealed reduced lower facial height, small rings under his eyes, and an everted nose. His skin and hair were normal (Figure 4). Intraoral clinical findings showed presence of severe oligodontia with only three primary teeth present in the upper arch. Upper central incisor was of irregular size and shape. Alveolar processes were thin, sharp, and undeveloped (Figure 4).

**Figure 5:**
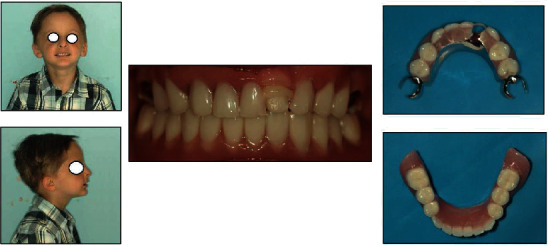
Extraoral and intraoral examination after the delivery of the prosthesis.

Treatment involved construction of interim prosthetic appliances to achieve increase of the vertical dimension and reclamation of the lower facial height. A removable partial denture that extended up to the first primary molars was constructed for the maxilla with metallic clasps on second primary molars and an acetal clasp on the primary central incisor. A complete removable denture, extending up to the second primary molar region, was constructed for the mandible (Figure 5).

## 4. Case Three

A 3.5-year-old Caucasian male was referred to our clinic by a paediatric dentist for prosthodontic management. The patient had been diagnosed with ED with the family history revealing an x-linked heritage, with the mother being carrier of the same gene.

**Figure 6:**
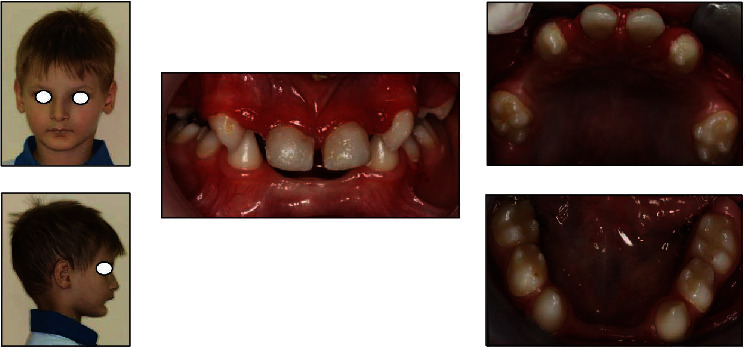
Initial extraoral and intraoral examination.

Extraoral examination (Figure 6) revealed a short lower facial height, maxillary protrusion, acute labiomental fold and an inverted upper lip. Patients' hair were thin and sparse, his eyelashes and eyebrows scant, and his skin soft, thin, and dry. Intraorally, six primary teeth were present in each arch, incisors had composite build-ups, and primary molars had been restored (Figure 6).

**Figure 7:**
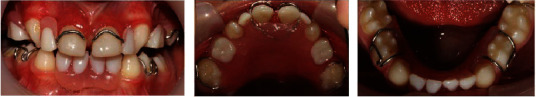
Intraoral examination after treatment completion.

Treatment involved construction of interim removable partial dentures to replace missing teeth in both arches. For better retention, the upper denture had Adam's clasps on second primary molars and wrought-wire clasps in central incisors, while lower denture only had Adam's clasps in first primary molars (Figure 7).

## 5. 2-Year Follow-Up

For all cases, follow-ups were performed every 6 months during whichprostheses were adapted, according to patient's growth pattern. Patients' acceptability and satisfaction were evaluated with three simple questions regarding mastication, aesthetics, and overall self-esteem. Answers were given in a five-item image scale ranging from “not at all” to “very much.”

**Figure 8:**
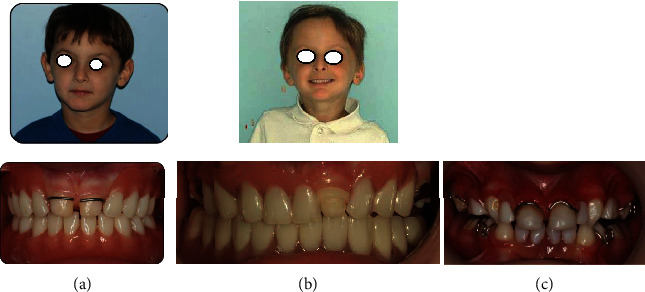
Examination at 2-year follow-up for (a) case 1, (b) case 2, and (c) case 3.

At the 2-year follow-up (Figures 8(a)–8(c)), treatment was considered successful as oral hygiene was good, no new carious lesions were recorded, and occlusion was stable. Patients were very pleased and had fully adopted to the prostheses, and satisfaction with function and aesthetics was reported by both the patients and their parents.

Evaluation of further development of the craniofacial complex and eruption of permanent teeth will be performed periodically for all three patients, and future prosthodontic management will be modified according to the growth of the patients and the number of existing permanent teeth, evaluated after the age of 6 with an orthopantomogram.

## 6. Discussion

This is a report of three very young children with oligodontia due to ED attempting to stress the challenges of oral rehabilitation at this age, with interdisciplinary approach. The aim of the treatment was replacement of missing teeth to improve aesthetics and address the parent's chief complaint, as well as restoration of vertical dimension in order to improve mastication and preserve the alveolar ridges.

In the literature, there is no recommendation of aspecific age for treatment initiation in patients with ED nor guidelines indicating a timeline for radiographic examination. As stated in the revised guidelines by the European Academy of Paediatric Dentistry, “good cooperation of the patient, including the ability to follow instructions and to remain still for the required exposure time, has to be established as a minimum requirement to ensure an appropriate image quality. This can be expected in young patients around the age of 4 years at the earliest”. [17] For this reason, we chose not to perform a radiographic examination since at present time and stage of development, it would not directly influence treatment planning. Nevertheless, and as patients approach mixed dentition, an orthopantomogram will be necessary to report on permanent teeth agenesis and plan future prosthodontic management.

According to Pigno et al. [18], prosthetic rehabilitation should be first considered around the age of 3-4 years, before the child goes to school. Children and adolescents with ED often express anxiety for their external appearance and shape of teeth, rendering early treatment necessary [5]. High levels of trust and good communication between children and the clinician are very important for completing treatment, especially when the children are very young. Patients and their parents should be included in the decision upon rehabilitation process, and they should be informed in advance about any possible risks, expected therapeutic outcomes, and financial requirements since the latter is a lifetime burden [3, 18, 19]. Nevertheless, very young children, as in our cases, especially pre-schoolers , have no issues with function and appearance, and the parents are the ones who seek dental care, mostly for aesthetic reasons. Therefore, the biggest challenge in these cases derives from the different perspectives for the treatment outcome between the patient and their parents and the clinician.

Prosthetic rehabilitation, with removable partial or complete dentures, as in our cases, is among the most common treatment modalities, especially in very young patients [20–22]. They provide a non invasive, reversible, economical substitution of missing teeth with ease of application and repair while allowing jaw growth through regular adjustments of the prostheses every 2-4 years [2, 5]. In addition, they are often preferred for early treatment in very young children to allow adequate skeletal growth before implant placement [5]. However, they may present reduced retention and stability due to the irregular shape of the teeth, the “knife-edge” morphology of the alveolar ridges, and the underdevelopment of salivary glands, which leads to mucosal dryness [3, 5, 23]. To overcome retention problems, composite build-ups were made in the conical-shaped teeth in all presented cases which also improved aesthetics. Furthermore, clasps were added to the prosthesis to enhance retention and stability overcoming issues due to alveolar ridge morphology. Clasp selection for the partial dentures was determined according to the position and shape of the present teeth. .

A major problem with partial removable prostheses is maintenance of adequate oral hygiene and integrity of the remaining teeth. This underlines the importance of a good preventive program that actively involves the parents and which should be regularly reinforced and modified accordingly, as the child grows. The preventive program should always include regular tooth brushing at least twice a day with fluoridated toothpaste and with appropriate fluoride content, according to patient's age. Dental prostheses also require proper cleaning to prevent the development of stomatitis and fungal infections in the oral cavity. Parental supervision and assistance is required as patients present incomplete development of muscle co-ordination, due to their young age and therefore are unable to reciprocate to proper brushing requirements. In addition, diet adjustments with limited consumption of sugary drinks and foods between meals reduce the risk of developing caries on the teeth present.

Alternative rehabilitation choices that have been reported in the literature include overdentures and fixed partial dentures. Overdentures provide a more retentive result with the advantage of preserving the alveolar ridge height. However, they require the presence of sufficient number of teeth and have the downside of extensive tooth preparation. Fixed partial dentures also render a more stable and aesthetic result, but they are avoided in young patients due to the risk of affecting jaw growth, especially when crossing the midline [2]. Lately, devices comprising of two parts, one removable and one fixed, have been reported, allowing dentofacial growth and ensuring increased stability of the prostheses [23]. None of the above could be performed in any of our cases, mainly due to the increased number of missing teeth.

Lately, placement of implants, even in young children with hypodontia, has been discussed in the literature, since this is a more stable and aesthetic solution, compared to conventional prostheses, provided that there is sufficient bone volume. The ideal time of implant placement is defined by child's dental and skeletal maturity and is mostly indicated after the completion of jaw growth, to avoid potential trauma in the adjacent tooth germs and possible interference with the eruption of permanent teeth [6, 24, 25].. However, there are cases of ED with implant rehabilitation at 5-6 years of age [4, 6, 18, 26–28]. It is an expensive treatment modality and a challenging process, especially for young children, and implants can only be placed under general anesthesia. For all of the above reasons, implants were not considered as a treatment option in the cases of the present report.

ED patients overall exhibit good adaptation to removable prostheses [3, 18, 20–22], and this is in accordance with the present cases, where patients exhibited total adjustment to the prostheses, despite their very young age. Rehabilitation with removable prostheses is associated with improved OHRQoL, in older children and adolescents [29–31]. There is one case report by de Alencar et al. [32], where early rehabilitation with removable prostheses showed improved OHRQoL of a preschool child, as assessed by the parents. Children have high adaptability, and therefore, it is no surprising that soon after the delivery of the prostheses, these patients reported no speech or masticatory difficulties even in consumption of solid and hard food.

Interdisciplinary approach, by a group of specialists, contributed to the fast adaptation and satisfaction of the children and their families, ensuring quality of treatment while lessening the probability of any functional or aesthetic implications. Rehabilitation of patients with ED, especially those of younger ages, is a challenging process with long-term impact on patients' quality of life; therefore, co-ordination between clinicians is necessary for optimum results. The role of the paediatric dentist is of paramount importance especially when treatment is provided without pharmacological behavior management techniques as in the cases presented here. Although rehabilitation was complicated, proper behavior guidance led to successful results, establishing the basis for future dental management.

## 7. Conclusions

1. Rehabilitation of oligodontia may be challenging due to accompanying oral findings, dentofacial growth considerations, and behavioral issues.
2. In very young patients, the biggest challenge for oral rehabilitation derives from the different perspectives for the treatment outcome between the patient, the parents, and the clinician.
3. Establishment of good rapport between the patients, parents, and clinician is the key for the success of the treatment even with the use of nonpharmacological behavioral management techniques.
4. Removable prosthesis provides a non invasive, relatively affordable treatment option with ease of application and repair while allowing jaw growth through regular adjustments.
